# Delay in diagnosis to treatment and impact on survival of gastric adenocarcinoma in a low income setting without screening facility

**DOI:** 10.1038/s41598-023-47415-y

**Published:** 2023-11-23

**Authors:** D. Subasinghe, P. K. B. Mahesh, G. K. Wijesinghe, S. Sivaganesh, A. Samarasekera, M. D. S. Lokuhetty

**Affiliations:** 1 Department of Surgery, Faculty of Medicine, University of Colombo, University Surgical Unit, The National Hospital of Sri Lanka, Colombo, Sri Lanka; 2 Postgraduate Institute of Medicine, University of Colombo, Colombo, Sri Lanka; 3 Department of Pathology, Faculty of Medicine, University of Colombo, Colombo, Sri Lanka; 4 Department of Pathology, National Hospital of Sri Lanka, Colombo, Sri Lanka

**Keywords:** Gastroenterology, Health care, Medical research, Oncology, Risk factors, Signs and symptoms

## Abstract

The treatment modality of gastric adenocarcinoma (GCA) depends on the stage of the disease at the clinical presentation. Long delays are probably an unfavorable factor for the patient's prognosis. A prospective longitudinal, study involving 145 consecutive GCA was conducted at the National Hospital of Sri Lanka (NHSL). The overall delay (in weeks) was recorded for each patient and divided into four periods-patient, endoscopy, pathology and treatment. The median and Interquartile Range (IQR) duration of delays were calculated and differences were explored with chi square test and Mann Whitney *U* test Survival analysis was done with Kaplan Meier technique and Cox regression. The median duration of delays for patient, endoscopy, histology reporting delay, other histology delay (specimen transfer delay and report receipt delay) and treatment were 18 (IQR 14–27), 2 (IQR 2–3), 3 (IQR 2–3), 2 (IQR 1–2) and 6 (IQR 4–8) weeks respectively. Delayed patient presentation to hospital was associated with significant adverse median survival 16 (IQR 11.5–22.5) weeks versus 20 (IQR 16–27.5) weeks, p = 0.004. Delay in initiating treatment was associated with significantly lower median survival 04 (IQR 4–6) weeks versus 06 (IQR 4–8) weeks, p = 0.003. Over 60% of both proximal and distal GCA presented at an advanced radiological stage (stage III/IV). The Kaplan Meier analysis showed that the higher hazard function was associated with a higher tumour stage and undergoing chemotherapy. Age of the patient and the treatment modality were significant predictors of the survival. Patient delay and delay in initiation of definitive treatment are the most important factors that adversely affect the outcomes of GCA. Public health interventions aiming to shorten the patient delay time with proper referral for specialist care would play an important role. Also, it is important to minimize these preventable delays and there should be time limits in producing the histopathology report and to establish online portals of hospital and laboratory information systems for easy access of histology reports in future.

## Introduction

Gastric adenocarcinoma (GCA) is one of most common neoplasms in the world with a significant impact to the patient and to the health system. It is the third leading cause of death due to cancer worldwide^1^. Eastern and central Asian regions are associated with the highest estimated mortality rates^2,3^. According to the National Cancer Registry data of 2020, the age standardized incidence rate per 100,000 males was 4.4 and for females was 1.7^4^ The National Cancer Registry 2020 represents a total number of 726 cases (male = 497, females = 229)^4^. This might not reflect the true incidence of the disease due to potential under-reporting of cases to the national government statistic processes from the private medical care and from patients opting for alternative or traditional forms of treatment.

The treatment modality of GCA depends on the stage of the disease at the clinical presentation. Prognosis of GCA remains dismal with a 5-year survival being around 5–20%, despite advancement of endoscopic, surgical and oncological modalities^5,6^. Therefore; it remains a poorly resolved oncological problem. The main reason behind this is the advanced stage of tumours at presentation. Hence, long delays are probably an unfavorable factor for the patient's prognosis. Overall in literature, only few studies have looked^7,8^ into the aspects of delay in the diagnosis of GCA.

In Japan, the screening programme for GCA resulted in 5-year survival rates above 60%^9,10^. Given the low incidence of disease^4^, in the Sri Lanka setting, implementation of a comprehensive screening programme is not feasible. Therefore, diagnosis of GCA inevitably follows symptoms reported by patients. According to available published data in the Sri Lankan population, majority presented at a very advanced stage at the time of diagnosis^11–13^. Therefore identification and intervening factors by minimizing delay diagnosis and treatment of GCA benefits in terms of improved surgical treatment, quality of life and survival.

In this setting, only around 40% of patients with GCA qualify for curative treatment, which consists of surgical resection with or without adjuvant chemotherapy^11^. The 5-year overall survival (OS) after curative treatment remains poor^11^. Overall HER2 positivity in Sri Lanka GCA was found to be relatively lower according to a previous prospective study^11^. Therefore, exploring factors that result in advance presentations leading to poor outcome are a timely need for improving survival of GCA in this setting.

At present there are no published data regarding the delayed presentation of GCA and a possible association between symptom-to-treatment delays or the stage of the GCA at the time of treatment and its impact of survival in relation to the local setting. This study was planned with the aim of bridging this gap of evidence.

## Methods

### Study design, study setting, study population and ethics

This prospective longitudinal, study was conducted at the National Hospital of Sri Lanka (NHSL) which is the premier multi-specialty tertiary care center in the country. Ethical approvals were obtained from the ethics committees of the Faculty of Medicine, University of Colombo (EC 11–139) and the National hospital of Sri Lanka (AA/ETH/2013). One hundred and forty-five (145) consecutive GCA patients presenting to the NHSL over four years (2012–2016 April) were studied and followed up till 2017 December. All patients underwent upper gastrointestinal endoscopy (UGIE) and biopsy for diagnosis. All underwent contrast enhanced computerized tomography (CECT) of the abdomen and thorax for radiological staging and radiological data were documented using a structured data sheet. Radiological data were used to determine the T (tumour), N (nodal with enlargement > 1 cm)^13^ and M (metastasis) stages of patients who only had biopsies without resections. Pathological data were used to determine the T and N (nodal metastasis) stages in patients who underwent resections. The TNM stage was determined in accordance with the seventh edition of the UICC guidelines which was in use during the period of study^14^.

All patients (aged ≥ 18 years) with GCA were included in the study. Exclusion criteria included; gastro-oesophageal junctional (GOJ) tumours and patients with other rare histological types of gastric cancers; such as adenosquamous carcinomas, undifferentiated carcinomas, neuroendocrine carcinomas, gastric lymphomas and gastrointestinal stromal tumours (GISTs).

An interviewer administered questionnaire that included basic demographic data, onset of symptoms, type of symptoms, time of visit to hospital, time of referral to specialist and endoscopy performance, location of gastric tumour, pathologic confirmation and time of surgery/palliative chemotherapy was administered. Patients were recruited on daily basis during study period of 2012–2016. All patients were followed up via telephone interview 3 monthly.

Distal GCA was defined as tumours beyond the region from the incisura angularis to the antrum-pylorus. Anorexia and weight loss, gastric outlet obstruction (GOO), abdominal mass were considered as advanced symptoms. Survival data of GCA patients were obtained by interviewing patients at the follow up clinic, contacting the participant/relatives via available contact information when a participant was not present for the scheduled clinic follow up.

### Data definitions

The date of diagnosis was defined as the date of the first gastrointestinal endoscopy as a proxy date of diagnosis, on which the biopsy sample was obtained to diagnosis GCA by histological examination. The waiting time (WT) was defined as the interval between the date of diagnosis and the date of surgery for the curative treatment group, and as the interval between the date of diagnosis and the date of initiation of palliative chemotherapy for advanced stage GCA group.

The overall delay (in weeks) was recorded for each patient and divided into four periods: (1) Patient delay—the time from first symptoms to first visit to doctor, (2) Endoscopy delay—the time from first visit to hospital to endoscopy, (3) Pathology delay—the time from endoscopy performance to establishment of definitive histological diagnosis by receipt of report to wards and (4) Treatment delay—the time from histological diagnosis to the definitive treatment (surgery/palliative chemotherapy). Pathology delay was further analyzed as specimen transfer delay, histopathology reporting time and pathology report receipt delay. Specimen transfer to the lab and report dispatch to surgical wards from the laboratory, occurs physically in this setting, therefore specimen transfer delay and pathology report receipt delay were defined as ‘other pathology delay’ after excluding histopathology reporting time.

### Statistical analysis and outcome measures

The median and Interquartile Range (IQR) were used to express the delays in relation to different aspects. The main outcome measures were the patient delay, endoscopic delay, histopathology reporting delay, other pathology delay, treatment delay and 5 year survival.

The statistical significance of the difference of the delay between two groups (i.e. who survived for 5 years versus who did not, tumor stage I/II versus III/IV, proximal versus distal location, who underwent curative resection versus palliative chemotherapy) was evaluated with Mann Whitney *U* test. The association between the occurrences of advanced symptoms with definitive treatment was determined with Chi Square test. The significance level was considered as 5%. For survival analysis, Kaplan Meier hazard function curves were generated with stratifications for gender, tumour grade, tumour stage and treatment modality. Multivariable survival analysis was done with Cox regression. Age, gender, tumour stage, tumour grade, treatment delay, histopathology delay were entered as potential predictors for the Cox Regression analysis.

### Ethics approval and consent to participate

This study was performed in accordance with the Declaration of Helsinki. Ethical approvals were obtained from the ethics committees of the Faculty of Medicine, University of Colombo (EC 11–139) and the National hospital of Sri Lanka (AA/ETH/2013).Consent for publication-Not relevant. Informed written consent was taken for participation in this study.

## Results

### Demographic, clinical-radiological-pathological characteristics

A male predominance was observed with a male: female ratio of 1.6:1. The median age at diagnosis was 58 (IQR 50–65) years. Over 60% of both proximal and distal GCA presented at an advanced radiological stage (stage III/IV). The majority [72(49.7%)] were of Lauren’s intestinal subtype. Table 1 depicts demographic characteristics of GCA when stratified under tumour staging.

**Table 1:** Socio-demographic characteristics of the study population with GCA when stratified under the tumour staging (N = 145).

Demographic and pathological characteristics	Tumour stage (n, %)				Total
	I	II	III	IV	
Gender					
Male	8 (8.9)	26 (29.2)	14 (15.8)	41 (46.1)	89 (100.0)
Female	7 (12.5)	13 (23.2)	11 (19.6)	25 (44.7)	56 (100.0)
Age					
> 60	8 (10.0)	14 (17.5)	10 (12.5)	48 (60.0)	80 (100.0)
≤ 60	7 (10.8)	25 (38.5)	15 (23.1)	18 (27.6)	65 (100.0)

Based on clinico-radiological and pathological staging on biopsies and resections, most of the proximal tumours had been metastasized (stage IV, N = 41, 47.1%). The majority of patients [76 (52.4%)] underwent palliative chemotherapy, while only 69 (47.6%) patients underwent curative gastric resections. In patients who underwent curative resections, pathological staging was stage 1 (n = 15, 21%) stage II (n = 35, 50.7%), stage III (n = 17, 24.6%) and stage IV (n = 2, 2.9%). Table 2 shows the tumour location of this study population with GCA when stratified under the demographic and pathological characteristics.

**Table 2:** Tumour location and the demographic and pathological characteristics of study population (N = 145).

Clinico-demographic and pathological characteristics	Histological classification total (n, %)		
	Proximal (n, %)	Distal (n, %)	
Gender			
Male	53 (59.6)	36 (40.1)	89 (100.0)
Female	34 (60.7)	22 (39.3)	56 (100.0)
Age			
> 60	50 (62.5)	30 (37.5)	80 (100.0)
≤ 60	37 (56.9)	28 (43.1)	65 (100.0)
Tumour stage*			
I	7 (46.7)	8 (53.3)	15 (100.0)
II	25 (64.1)	14 (35.9)	39 (100.0)
III	14 (56.0)	11 (44.0)	25 (100.0)
IV	41 (62.1)	25 (37.9)	66 (100.0)
Tumour differentiation			
Well differentiated	26 (68.4)	12 (31.6)	38 (100.0)
Moderately differentiated	34 (60.7)	22 (39.3)	56 (100.0)
Poor differentiation	27 (52.9)	24 (47.1)	51 (100.0)

*Combined clinicopathological and radiological tumour staging.

### Delay in presentation, diagnosis and definitive treatment

The median duration of delays for patient, endoscopy, histology reporting delay, other histology delay (specimen transfer delay and report receipt delay) and treatment were 18 (IQR 14–27), 2 (IQR 2–3), 3 (IQR 2–3), 2 (IQR 1–2) and 6 (IQR 4–8) weeks respectively.

### Clinical presentations, tumour location, tumour stage, overall survival and delays

**Figure 1:**
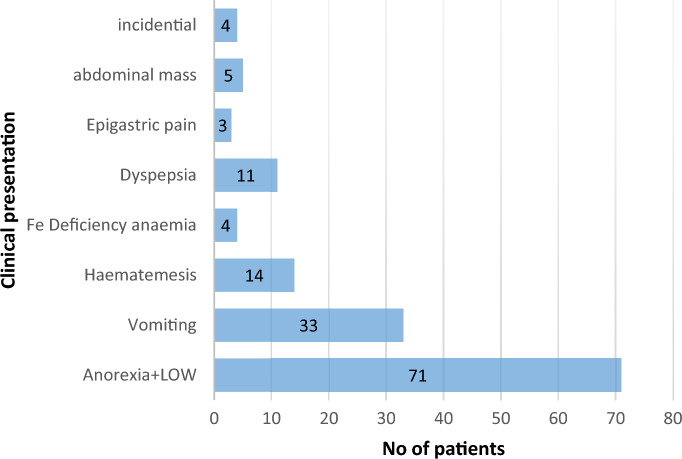
Distribution of types of clinical presentation.

Figure 1 depicts the clinical presentation of GCA. Out of the participating patients 75.1% (n = 109/145) were with advanced clinical presentations such as anorexia and weight loss, vomiting, abdominal mass.

**Figure 2:**
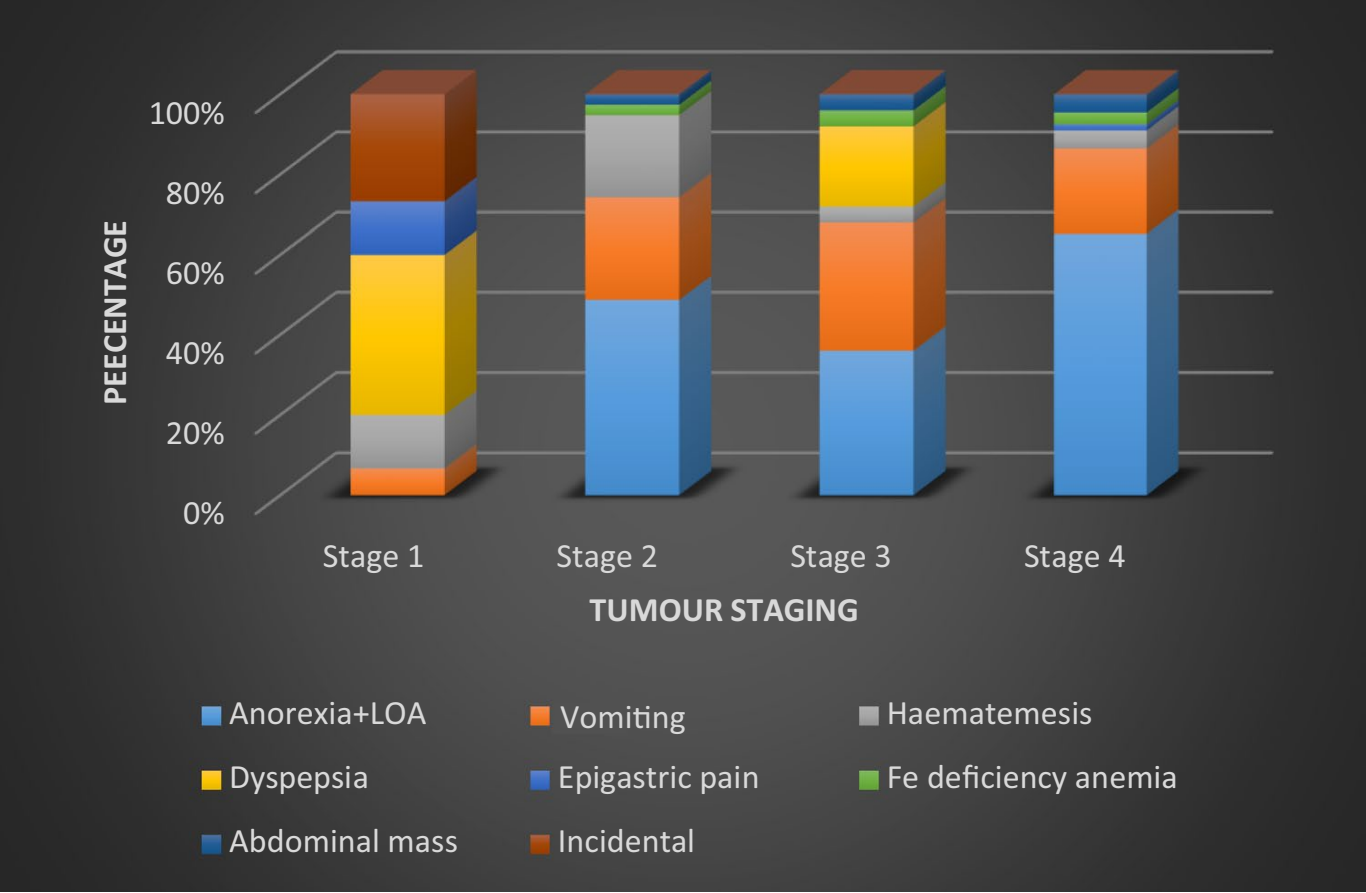
Distribution of eight clinical symptoms within each radiological stage.

As shown in Fig. 2, symptoms like anorexia + LOA were common in advanced stages whereas those which were like: dyspepsia, was found in earlier stages. Gastric outflow obstruction was seen in all stages but mainly across stage II–IV.

The median patient delay [20 (IQR = 15–28) weeks] in advanced clinical presentations was higher than other presentations (e.g. dyspepsia) which was 16 (IQR 11–22.5) weeks. The difference was statistically significant (p = 0.005). Patients with advanced clinical presentations had significant delay (p = 0.01) in initiating definitive treatment compared to those who did not have an advanced clinical presentation. The association between the occurrences of advanced symptoms with definitive treatment is shown in Table 3.

**Table 3:** The association between the occurrences of advanced symptoms with definitive treatment.

Clinical presentation	Definitive treatment		Total (%)	Association
	Palliative chemotherapy	Curative resection		
With advanced symptoms	64 (58.7)	45 (41.3)	109 (100.0)	χ^2^ = 6.99 P = 0.008 OR = 2.84 CI = 1.29–6.27
Without advanced symptoms	12 (33.3)	24 (66.7)	36 (100.0)	
Total	76	69	145	

Table 4 shows the associations between overall survivals with different types of delays in GCA patients with delayed patient presentation to hospital and delay in initiating treatment being associated with significant adverse median survival (p < 0.05). There were significantly higher median delays in patient and treatment components in relation to advanced tumour stages (Stage III/IV) when compared with early stage GCA (Stage I/II). The opposite association was seen for other histology delay. There was no significant association between delay analyses versus the tumour location in this cohort of patients with GCA (Table 4).

**Table 4:** Overall survival in relation to delays and tumour characteristics.

	Patient delay	Endoscopy delay	Histology pathology reporting delay	Other pathology delay	Treatment delay
Live at the end of 5-year follow up period					
Yes	16.0 (11.5–22.5)	2.0 (2.0–3.0)	2.0 (2.0–3.0)		4.0 (4.0–6.0)
No	20.0 (16–27.5)	2.0 (1.0–4.0)	3.0 (2.0–3.0)		6.0 (4.0–8.0)
Significance	P = 0.004*	P = 0.827	P = 0.055		P = 0.003*
Tumour stages					
Stage (I/II)	16 (11.75–22)	2 (1.75–3)	2 (2–3)	2 (2–3)	05 (4–6)
Stage (III/IV)	20 (15–28)	2 (2–4)	3 (2–3)	1 (1–2)	07 (4–8)
Significance	P = 0.002*	P = 0.168	P = 0.077	P < 0.001*	P = 0.001*
Location of the tumour					
Proximal	17 (13.0–26.0)	2 (2–3)	2 (2–3)	2 (1–2)	06 (4–8)
Distal	18 (14–27)	2 (1–3)	3 (2–3)	2 (1–2)	06 (4–6.5)
Significance	P = 0.417	P = 0.470	P = 0.787	P = 0.406	P = 0.425

*Statistically significant.

Table 5 depicts associations of treatment outcomes of GCA with waiting time.

**Table 5:** Associations of treatment outcomes of GCA in relation to waiting time.

	Curative resection	Palliative chemotherapy	Association
Waiting time-median (IQR)	11 (10–14.75)	14 (10–13)	P < 0.0001*
Histopathology reporting time-median (IQR)	2 (2–3)	3 (2–3)	P = 0.497
Other pathology delay-median (IQR)	2 (2–2.5)	1 (1–2)	P = 0.037*

*Statistically significant.

Longer waiting times (WT) was associated with adverse outcomes of GCA (Table 5). There was a statistically higher “other pathology delay” (which includes specimen transfer delay and pathology report receipt delay) of those who underwent curative resection compared to those who underwent palliative care (p = 0.037).

The distribution of eight clinical symptoms within each radiological stage is shown in Fig. 2.

The Kaplan Meier analysis showed that the higher hazard function was associated with a higher tumour stage and undergoing chemotherapy (i.e. compared to undergoing surgery). The hazard function curves are shown in supplementary figures (Figs. S1–S4).

Cox regression is shown in Table 6. Higher hazard ratios were associated with those with advancing age and those who received chemotherapy (compared to those who underwent surgery). Once adjusted to the age, gender, tumour grade, tumour stage and treatment modality, the treatment delay and the histopathology delay were not significantly associated with the survival.

**Table 6:** Beta coefficients and hazards ratios obtained with Cox regression.

Variable	Beta	Significance	Exponential beta and confidence interval
Advanced age^a^	0.122	P < 0.001	1.13 (1.08–1.18)
Male gender^b^	− 0.220	P = 0.366	0.80 (0.49–1.30)
Low tumour grade^c^	0.140	P = 0.566	1.15 (0.71–1.86)
Tumour stage I or II^d^	0.058	P = 0.905	1.06 (0.41–2.74)
Treatment modality-chemotherapy^e^	0.949	P = 0.041	2.58 (1.04–6.41)
Treatment delay^a^	− 0.032	P = 0.321	0.97 (0.91–1.03)
Histopathology delay^a^	− 0.126	P = 0.318	0.88 (0.69–1.13)

^a^Entered as a numerical variable.

^b^Baseline—female gender.

^c^Baseline—High tumour grade.

^d^Baseline—tumour stage III or IV.

^e^Baseline—treatment modality-surgery.

## Discussion

This Sri Lankan study explored into components of delay in the diagnosis and treatment of GCA. It showed that delayed patient presentation to hospital and delays in initiating definitive treatment were potentially adversely associated with the survival. The study additionally revealed that patients with advanced clinical presentation had delays in getting the definitive treatment initiated. GCA patients presenting with advanced symptoms, had more chance of developing incurable disease requiring palliative chemotherapy, than getting a curative surgical resection. It also identified that advanced tumour stages (Stage III/IV) were associated with higher median delays in seeking medical advice and these patients had significant pathology delay (due to longer specimen transfer and report dispatch times) and delay in initiating treatment as well.

This information is invaluable and has to be addressed in future, with a view to improve the outcomes of GCA in Sri Lanka and in similar settings. The waiting time is considered an important quality indicator for cancer care^8,15^. This study showed that a longer waiting time is associated with significant chances of being allocated for palliative chemotherapy due to advanced stage of disease, and with worse survival outcomes. The longer waiting times are known to negatively influence patients’ quality of life, resulting in psychological distress, and poor oncological outcomes in various cancers^8,15,16^. In the diagnostic pathway of patients with GCA, the patient delay is the longest delayed component in this cohort. Overall delays in endoscopy and pathology were relatively shorter.

An Iranian study^17^ involving 63 patients, revealed that median patient, endoscopy, pathology, surgeon delays were 1.1, 8.1, 1.7 and 1 weeks respectively. In a study by Tata et al.^18^ median patient delay was 15.23 and endoscopy delay was 3.37 weeks. Both these studies^17,18^ had included a mixture of GCA and GOJ tumours. In a study^19^ on both oesophageal and GCA in Netherlands found that delays involving primary care interval to be, 12 days (interquartile interval 1–43), secondary care interval: 13 days (interquartile interval 6–29) and diagnostic interval: 31 days (11–74). In contrast, in the present study had higher median duration of delays for patient, endoscopy, pathology, definitive treatment and were 18 (IQR 14–27), 2 (IQR 2–3), 4 (IQR 4–5) and 6 (IQR 4–8) weeks respectively.

Sinister symptoms like weight loss^20–22^, palpable abdominal mass^21,23,24^ have been identified as independently-related adverse factors that result in fatal outcome in GCA. The majority of our patients presented with advanced stage with sinister symptoms precluding curative resection. Hence, they were referred for palliative chemotherapy. Therefore; GCA patients presenting with advanced symptoms, had more chance of developing incurable disease requiring palliative chemotherapy, compared to those who received curative surgical resection.

Prognosis of GCA is highly dependent on disease stage at diagnosis^25^. Surgery, is the mainstay of treatment that could only cure some patients with early-stage disease^26^. To date, the survival rates of GCA, as well as the differences in survival rates observed between Eastern and Western GCA, have been mainly attributed to the TNM stage^25^. Therefore, an efficient diagnostic pathway is the key to timely diagnosis. In the diagnostic pathway of patients with GCA, the patient delay is the longest, followed by delay in initiating definitive treatment either surgery or chemotherapy. In contrast, delays in performing endoscopy and pathology delays are shorter. Findings of the present study indicate that patients may not be fully aware of alarm symptoms, since durations of the patient delays were long. Raising the awareness of GCA alarm symptoms in susceptible population may be the most efficient way to improve prompt presentation to health services and shorten the time from the development of symptoms to diagnosis. On the other hand, exploring more into the reasons for postponing healthcare consultation would enable designing a targeted approach to the problem.

Thus interventions can be integrated into the well-developed public health infrastructure in Sri Lanka, under which each household is allocated into a geographical public health unit called as Medical Officer of Health (MOH) area. The MOH staff deliver public health services in clinic-based settings as well as visiting the houses as domiciliary-services. Hence if a set of health education advices can be developed targeting the GCS alarm symptoms, those messages could be delivered to the community through these staff members who arrange the public health services according to the life-cycle approach.

Although a screening endoscopy program for upper gastrointestinal (GI) cancers is unavailable in Sri Lanka, the upper GI endoscopy facilities have much improved over the past decade. Despite ample access to upper GI endoscopy, delays in diagnosis still seem to be common and virtually affect the stage of GCA at diagnosis, as well as outcome of the patients. In fact, in a country like Sri Lanka, where a screening programme is not feasible due to the lower incidence of the disease, diagnosis of GCA inevitably relies on symptoms reported by patients. Furthermore, better collaboration between curative and preventive sector is the way forward to improve outcomes in GCA. Therefore, increased awareness of GCA symptoms by the susceptible population, as well as correct interpretation of the symptoms and prompt referral for endoscopic investigation, could reduce the diagnostic delay and, theoretically, improve survival. Once method of intervention in this regard could be the formulation of guidelines aiming to facilitate General Practitioners to promptly refer these patients for specialized care.

In the present study there were higher median delays in seeking medical advice in patients having advanced tumour stages (Stage III/IV). These belonged to the group of patients who had significant pathology delay confirming the diagnosis, and delay in initiating treatment. Pathology results are critical for the diagnosis and surgical decision making regarding GCA. In the palliative group of GCA, tissue diagnosis with pathology report is a key factor in initiation of chemotherapy. The present study showed that specimen transfer delay to lab and delay in receipt of the pathology report to the ward mainly contributed to overall pathology delay. Therefore, it is important to minimize these preventable delays and there should be time limits in producing the histopathology report. It is also important to establish online portals of hospital information system (HIS) and laboratory information system (LIS) for easy access of histology reports in future with in our hospital system. Furthermore, one method of intervention isto make sure that specimens are immediately transferred to the laboratory at the end of resection in theater or at the end of endoscopy list.

According to Cox regression analysis, age of the patient and the treatment modality, were significantly associated with the survival of the cohort. Patients who had palliative treatment (biopsies) had a higher hazard ratio compared to those who underwent surgical intervention which is plausible with their advanced disease profile. There was no significant association between histopathology, treatment delay with the hazard function. This could be due to other unrecognized contributing factors which need to be investigated with a larger sample.

Improving delay with the survival by timely detection of GCA among patients without alarm symptoms is challenging, given the high incidence of common upper GI symptoms and functional disorders at low risk of cancer. On the other hand, simply lowering the threshold for endoscopy is not the solution for reducing the time to referral as there is already a growing demand for diagnostic endoscopy services in secondary care. Lowering the threshold for endoscopy might result in increased risk of non-indicated endoscopies with normal results. Therefore, early referral of patients with alarm symptoms (i.e. anorexia, anaemia) without further delay is an important aspect in early diagnosis.

In Sri Lanka, despite the improvement of endoscopic facilities, delays in diagnosis still seem to be common. The patient delay and delay in initiating definitive treatment are the most important contributors to waiting time in GCA in this setting. This might affect the stage of GCA at diagnosis as well as outcome. Therefore, the increased awareness of symptoms, as well as correct interpretation of the symptoms and prompt referral for investigation, could reduce the diagnostic delay and, theoretically, improve survival.

This study is with several limitations. The factors which resulted in delayed presentation of patients to healthcare services as well as factors that resulted in delay in starting palliative chemotherapy such as patients’ wishes on alternative treatment strategies, beliefs, fears on initiating chemotherapy etc. were not investigated. Getting an insight in to these reasons for postponing medical consultation would be required for a targeted approach. Secondly, in patients who had delays in having primary resections, the exact reasons and possible causative factors such as postponement due to lack of adequate intensive care beds were not evaluated. The survival follow-up time was also not uniform and was in the range of 0–240 weeks. However as the exposures except for the “duration of chemotherapy” did not change in the follow up period, authors are confident that a major degree survival bias can be excluded. The “duration of chemotherapy” is usually decided based on case-by-case basis. The study only explored the patients visiting the government setting and did not explore those visiting the private sector.

## Conclusions and recommendations

Patient delay and delay in initiation of definitive treatment are the most important delays in GCA in our setting in Sri Lanka, where a comprehensive endoscopy screening programme for GCA is not available. Public health education with predominantly a community focus on susceptible population needs to be initiated and strengthened. Public health interventions aiming to shorten the patient delay time with proper referral for specialist care would play an important role. Formulation of guidelines aiming to facilitate General Practitioners to promptly refer these patients for specialized care is one important measure to overcome patient delay. It may be of interest to see whether these findings are applicable to similar low incidence settings for GCA with limited screening facilities.

### Supplementary Information

Supplementary Figures.

## Data Availability

All data generated or analysed during this study are included in this published article. Data is available for further analysis by an interested third party.
