# Supplementary material for: Delay in diagnosis to treatment and impact on survival of gastric adenocarcinoma in a low income setting without screening facility
Source: Sci Rep. 2023 Nov 23;13:20628. doi: 10.1038/s41598-023-47415-y (PMC10667260; doi:10.1038/s41598-023-47415-y)
Supplement: Supplementary file 1 — Supplementary Figures. [file 41598_2023_47415_MOESM1_ESM.docx]

**Kaplan-Meier**

| **Overall Comparisons** | | | |
| --- | --- | --- | --- |
|  | Chi-Square | df | Sig. |
| Log Rank (Mantel-Cox) | .677 | 1 | .411 |
| Test of equality of survival distributions for the different levels of Gender. | | | |


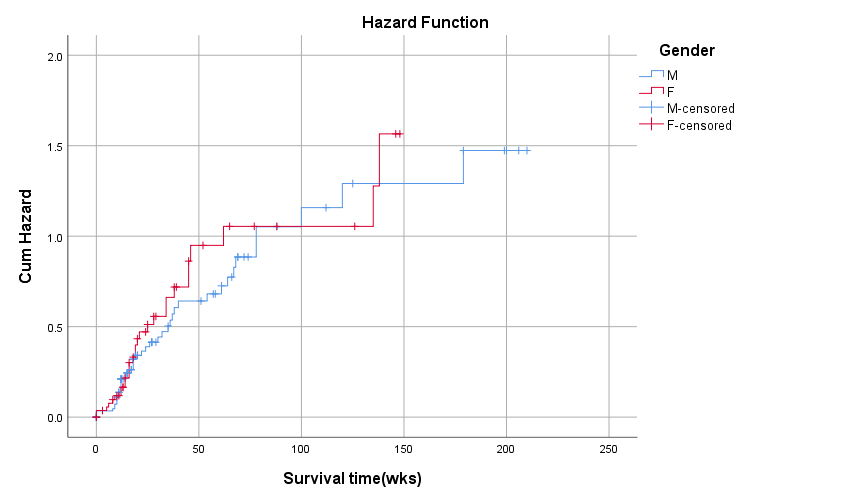


Figure S1: Hazard plot for the males (mentioned as M in the figure) versus females (mentioned as F in the figure) obtained with Kaplan Meier analysis

| **Overall Comparisons** | | | |
| --- | --- | --- | --- |
|  | Chi-Square | df | Sig. |
| Log Rank (Mantel-Cox) | .219 | 1 | .639 |
| Test of equality of survival distributions for the different levels of Grade. | | | |


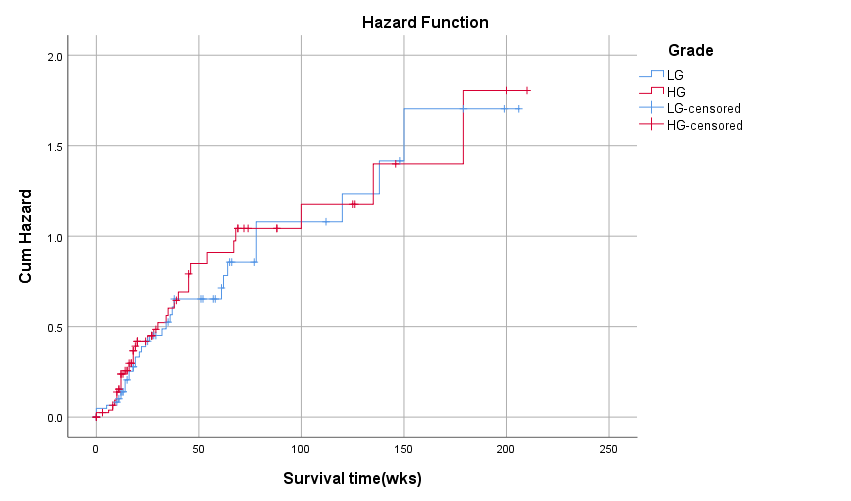


Figure S2: Hazard plot for the low grade tumours (mentiond as LG in the figure) versus high grade tumours (mentioned as HG in the figure) obtained with Kaplan Meier analysis

| **Overall Comparisons** | | | |
| --- | --- | --- | --- |
|  | Chi-Square | df | Sig. |
| Log Rank (Mantel-Cox) | 7.899 | 1 | .005 |
| Test of equality of survival distributions for the different levels of StageCAT. | | | |


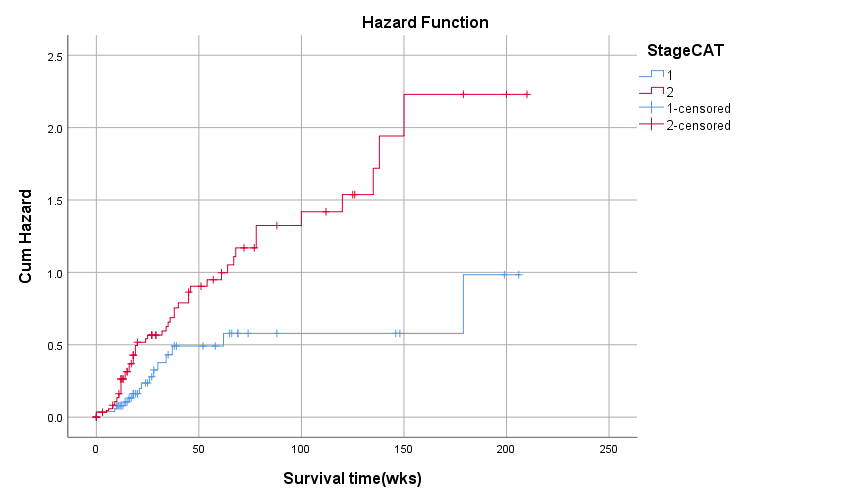


Figure S3: Hazard plot for the stage I/II (mentioned as StageCAT=1 in the figure ) versus III/IV (mentioned as StageCAT=2 in the figure ) obtained with Kaplan Meier analysis

| **Overall Comparisons** | | | |
| --- | --- | --- | --- |
|  | Chi-Square | df | Sig. |
| Log Rank (Mantel-Cox) | 15.794 | 1 | .000 |
| Test of equality of survival distributions for the different levels of Treatment modality. | | | |


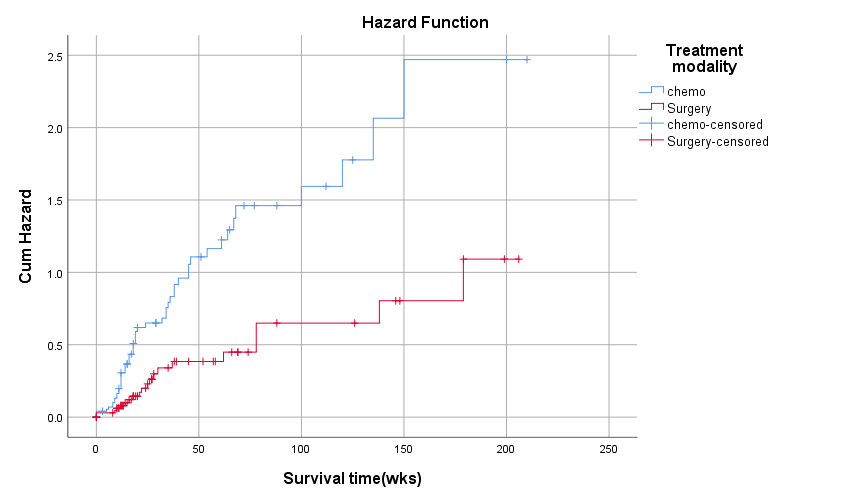


Figure S4: Hazard plot for the group undergoing surgery versus chemotherapy obtained with Kaplan Meier analysis
